# Cardiotoxicity After Anthracycline Chemotherapy for Childhood Cancer in a Multiethnic Asian Population

**DOI:** 10.3389/fped.2021.639603

**Published:** 2021-02-03

**Authors:** Varen Zhi Zheng Tan, Nicole Min Chan, Wai Lin Ang, Soe Nwe Mya, Mei Yoke Chan, Ching Kit Chen

**Affiliations:** ^1^Yong Loo Lin School of Medicine, National University of Singapore, Singapore, Singapore; ^2^Department of Family Medicine, Tan Tock Seng Hospital, National Healthcare Group, Singapore, Singapore; ^3^Cardiology Service, Department of Paediatric Subspecialties, KK Women's and Children's Hospital, Singapore, Singapore; ^4^Haematology-Oncology Service, Department of Paediatric Subspecialties, KK Women's and Children's Hospital, Singapore, Singapore; ^5^Department of Paediatrics, Yong Loo Lin School of Medicine, National University of Singapore, Singapore, Singapore; ^6^Khoo Teck Puat–National University Children's Medical Institute, National University Health System, Singapore, Singapore

**Keywords:** epidemiology, children, cardio-oncology, multiethnic, Asian population, heart failure, childhood cancer survivors, anthracycline cardiotoxicity

## Abstract

**Background:** Anthracyclines are widely used to treat childhood cancers; however, they cause cardiotoxicity. To address the paucity of clinical data from Asian populations, this study investigated the epidemiology of pediatric anthracycline-induced cardiotoxicity, during and after chemotherapy, in a multiethnic Asian population.

**Procedure:** This was a single-center, retrospective analysis of 458 anthracycline-treated pediatric oncology patients at KK Women's and Children's Hospital, a tertiary children's hospital in Singapore from 2005 through 2015. We investigated cardiotoxicity (defined as left ventricular fractional shortening <28% on echocardiography) and its risk factors using univariate logistic regression as well as survival estimates through the Kaplan-Meier method to compare survival distribution between patients with and without cardiotoxicity.

**Results:** Over a follow-up period of almost 4 years, we found that 7% (32/458) of the cohort developed cardiotoxicity, with 37.5% (12/32) of these manifesting as clinical heart failure, whilst the rest were asymptomatic. The cardiotoxic cohort demonstrated a significantly higher mortality rate compared to the non-cardiotoxic group at 46.9 vs. 19.2% (*p* < 0.001), of whom 3 (9.4%) died from end-stage heart failure. We found that traditional predictors such as female sex, age at diagnosis, and cumulative doxorubicin equivalent dose were not predictors of cardiotoxicity.

**Conclusion:** Our study reaffirms that freedom from symptoms does not ensure normal heart function and suggests that children with abnormal ventricular systolic function have higher mortality risk compared to those with normal systolic function. The findings contribute to improved understanding of the Asian burden to aid development of measures to prevent or reduce the risk of cardiotoxicity.

## Introduction

Pediatric cancer survival rates have risen significantly over the past few decades. (1) With contemporary therapies, over 80% of children diagnosed with cancer will become long-term survivors (2, 3). A key player that has contributed significantly to this improved survival in many childhood cancers is the use of anthracycline chemotherapy agents (e.g., doxorubicin, daunorubicin), which are administered to more than 50% of children with cancer. (4) However, anthracyclines have a well-known cardiotoxic effect, and although observed frequencies vary between studies, up to 57% of patients treated with an anthracycline may develop echocardiographic abnormalities (5). These abnormalities may be progressive in a significant proportion of patients (6–9). Childhood cancer survivors (CCS) exposed to anthracyclines have a significantly higher risk of developing cardiac disease compared to their siblings or age-matched peers (10–12). Cardiac disease is the leading non-oncological cause of premature death in CCS, with a 7-fold increased risk of premature cardiac death as compared to the general population. The relative risk of cardiac death remains elevated even in CCS who have survived for more than 25 years after their primary cancer (13–15).

The specific mechanism of cardiomyocyte injury from anthracyclines remains to be fully elucidated. While understanding the pathophysiology of the adverse effects is important in the development of preventive measures, recognizing the risk, and burden is the first crucial step toward developing strategies to promote cardiac risk prevention, detection, and management. Most available data on pediatric anthracycline-related cardiotoxicity were largely from Western populations (especially North America and Europe) but there is a paucity of clinical data from Asian populations (16), which may differ due to the presence of interethnic variations. In fact, recent pharmacogenomic studies have demonstrated that anthracycline-induced cytotoxicity may be attributable to a genetic component (17).

Consequently, it is essential to improve the understanding of the epidemiology of pediatric anthracycline cardiotoxicity in an Asian context. Here, we described the frequency and patient characteristics of adverse cardiac effects of anthracycline therapy, to better understand cardiotoxicity during and after initial chemotherapy for childhood cancers in multiethnic Singapore.

## Materials and Methods

### Study Design

This was a single-center, retrospective cohort study that was approved by the institutional review board with a waiver of informed consent. We identified children (<18 years of age) with cancer, who received anthracyclines as part of combination chemotherapy, diagnosed between 2005 and 2015 at the KK Women's and Children's Hospital (KKH), Singapore. Patients were screened for cardiotoxicity at the discretion of the treating oncologist due to the lack of an institutional protocol during the study period. Patients with cardiotoxicity were identified through the pediatric echocardiography database at KKH, from 2005 through 2016. These patients were censored at the date of last follow-up. Since this study sought to evaluate the cardiotoxic effects of anthracyclines, the date of last follow-up was defined as the date of latest echocardiographic study or the date of death.

Echocardiographic functional assessment measured left ventricular (LV) end-diastolic dimension (LVEDD), end-systolic dimension (LVESD), and fractional shortening (FS); biplane Simpson method for estimation of LV ejection fraction was not routinely performed, and advanced echocardiographic modalities (e.g., tissue Doppler, speckle tracking imaging) were not implemented prior to 2015. Cardiotoxicity was defined as abnormally low LV systolic function—LV FS <28%. Institutionally, we have opted to use FS for monitoring of ventricular function since it is widely used in various chemotherapeutic protocols for the monitoring of cardiotoxicity. For these children with cardiotoxicity, data regarding patient demographics, clinical characteristics, oncological diagnosis, anthracycline cumulative dose, echocardiographic parameters, and patient outcome were collected from patient medical records. The echocardiogram reports of patients with cardiotoxicity were retrieved and reviewed; cardiotoxicity was classified into acute (onset of cardiotoxicity during chemotherapy treatment), early (onset within 1 year of treatment completion) and late (onset > 1 year after completion of chemotherapy) cardiotoxicity (9). The cumulative dose of anthracycline was determined by conversion to doxorubicin isotoxic equivalents by multiplying the total anthracycline dose by 1 for doxorubicin and daunorubicin, 0.67 for epirubicin, 4 for mitoxantrone, and 5 for idarubicin (18). Anthracycline exposure was further classified as cumulative dose <250 mg/m^2^ (low dose) and ≥250 mg/m^2^ (high dose) (19). Cardioprotective agents such as dexrazoxane or other cardioprotective measures were not utilized during the study period.

### Statistical Analysis

Data were presented as median with 25th and 75th percentiles (interquartile range, IQR), and frequencies as appropriate. Comparison between patients with cardiotoxicity (Cardiotoxic Group) and those without cardiotoxicity (Non-cardiotoxic Group), as well as between patients in the ethnic subset of “Others” and those of the main ethnic groups (Chinese, Malay, and Indian) within the Cardiotoxic Group, were performed using Fisher's exact/chi-square test, or Kruskal–Wallis test. Univariate logistic regression was performed to explore the association between the primary predictor variables and cardiotoxicity as outcome, and odds ratio and 95% confidence intervals were calculated. A confidence level of *p* < 0.05 was considered statistically significant. Multivariable analysis was not conducted owing to the limited sample size, and in concordance to the paucity of significant results from the univariate analyses. The survival estimates were obtained using the Kaplan–Meier method; the log-rank test was used to compare survival distributions between the cardiotoxic and non-cardiotoxic groups. All statistical analyses were performed using Statistical Package for Social Science (SPSS) v23 (Chicago, IL, USA) and GraphPad Prism v8 (GraphPad Software, La Jolla California, USA).

## Results

### Study Population

During the study period, a total of 458 patients were newly diagnosed with cancer and received anthracyclines as part of their treatment protocol. Median age at diagnosis was 5.8 years (interquartile range, IQR 2.6–12.8 years); almost 40% of the patients were diagnosed at age <4 years. Table 1 presents the demographic and clinical characteristics of the study population, stratified by cardiotoxicity. There was a male preponderance (61.6%) in the total cohort which was not significant (*p* > 0.05). Commensurate with the demographics of Singapore, the majority of patients were ethnically Chinese (65.2%), followed by Malay (16.4%), Indian (10%), and others (8.3%), comprising of Caucasians, and foreign nationals, e.g., Filipino, Vietnamese etc. (20) More than half of the cohort (57.2%) was diagnosed with leukemia; lymphoma (18.3%) was the commonest solid tumor. Median cumulative doxorubicin isotoxic dose was 200 mg/m^2^ (IQR 200–300 mg/m^2^). One-third of the cohort received a cumulative dose of ≥250 mg/m^2^ (high dose). Three hundred and ninety-nine patients (399/458, 87%) had echocardiograms performed during chemotherapy, 323 patients (323/458, 70%) during the 1st year after completion of chemotherapy, and 347 patients (347/458, 76%) had long-term surveillance echocardiograms (more than 1 year after completion of chemotherapy).

**Table 1:** Demographics and characteristics of the study cohort.

**Characteristics**	**Overall (*n* = 458)**	**Cardiotoxic group (*n* = 32)**	**Non-cardiotoxic group (*n* = 426)**	***p*-value**
**Demographic**				
Sex				0.45
Female	176 (38.4)	10 (31.2)	166 (38.9)	
Male	282 (61.6)	22 (68.8)	260 (61.1)	
Race or Ethnicity				<0.001
Chinese	299 (65.2)	16 (50.0)	283 (66.5)	
Malay	75 (16.4)	6 (18.8)	69 (16.0)	
Indian	46 (10.0)	1 (3.1)	45 (10.7)	
Others	38 (8.3)	9 (28.1)	29 (6.8)	
**Clinical**				
Diagnosis				0.85
Leukemia	262 (57.2)	19 (59.4)	243 (57.0)	
Acute Lymphoblastic Leukemia (ALL)	220 (48.0)	11 (34.3)	209 (49.1)	
Acute Myeloid Leukemia (AML)	42 (9.2)	8 (25.0)	34 (7.9)	
Other tumors	196 (42.8)	13 (40.6)	183 (43.0)	
Neuroblastoma	36 (7.9)	5 (15.6)	31 (7.3)	
Osteosarcoma	44 (9.6)	4 (12.5)	40 (9.4)	
Lymphoma	84 (18.3)	2 (6.2)	82 (19.2)	
Ewing Sarcoma	17 (3.7)	1 (3.1)	16 (3.8)	
Hepatoblastoma	12 (2.6)	1 (3.1)	11 (2.6)	
Others	3 (0.7)	0	3 (0.7)	
Age at diagnosis, years	5.8 (2.6–12.8)	9.3 (2.7–13.1)	5.8 (2.6–12.8)	0.55
<1 year	33 (7.2)	3 (9.4)	30 (7.0)	
1–4 years	147 (32.1)	8 (25.0)	139 (32.6)	
>4 years	278 (60.7)	21 (65.6)	257 (60.3)	0.64
Cumulative doxorubicin equivalent dose, mg/m^2^	200 (120–300)	212 (120–315)	200 (120–300)	0.43
<250 mg/m^2^	310 (67.7)	17 (53.1)	293 (68.7)	
≥250 mg/m^2^	148 (32.3)	15 (46.9)	133 (31.3)	0.07
**Alkylating agents used**				
Cyclophosphamide	NA	12 (37.5)	NA	
Ifosfamide	NA	3 (9.4)	NA	
Radiation involving the heart	33 (7.2)	3 (9.4)	30 (7.0)	0.49
Follow-up duration, years	3.9 (2.1–9.5)	3.6 (1.11–11.8)	3.9 (2.2–9.3)	0.69
Mortality	97 (21.2)	15 (46.9)	82 (19.2)	0.001

*Values represent median (IQR) or number (percentage)*.

### Cardiotoxicity

Cardiotoxicity (defined as LV FS <28%) occurred in 32 of the 458 patients (7%) with median FS of 22.9% (IQR 18.6–26.4%). Table 2 summarizes the echocardiographic parameters of the group of patients with cardiotoxicity (cardiotoxic group). The median age of cardiotoxicity onset was 11.2 years (IQR 6.4–14.9 years), at an interval of 13.1 months (IQR 1.5–29.3 months) after completion of chemotherapy. Three patients (9.4%) had acute cardiotoxicity, 12 (37.5%) early cardiotoxicity, and 17 (53.1%) had late cardiotoxicity.

**Table 2:** Characteristics of the 32 study participants with cardiotoxicity (cardiotoxic group).

**Characteristics**	**No. of patients**	**%**
Age at diagnosis of cardiotoxicity, years	11.2 (6.4–14.9)	
Interval between anthracycline completion and cardiotoxicity, months	13.1 (1.5–29.3)	
**Onset of cardiotoxicity**		
Acute	3	9.4
Early	12	37.5
Late	17	53.1
**Echocardiographic parameters**		
LV FS, %	22.9 (18.6–26.4)	
LV FS *z*-score	−4.30 (−3.33 to −6.69)	
LVEDD, mm	44.7 (40.4–49.7)	
LVESD, mm	34.0 (29.4–39.9)	
**Cardiotoxicity treatment**		
Inotropic support	5	15.6
Diuretics	10	31.3
Angiotensin-converting enzyme inhibitors	14	43.8
Beta blockers	4	12.5
Mineralocorticoid receptor antagonists	6	18.8
Digoxin	9	28.1
**Mortality**		
All-cause mortality	15	46.9
Cardiac mortality	3	9.4
Follow-up duration, years	3.6 (1.11–11.8)	

*Values represent median (IQR) or number (percentage)*.

There was a male preponderance (22/32, 68.8%) in this cardiotoxic group. Commensurate to the demographics of Singapore, the majority of patients were ethnically Chinese; however, there was a significantly higher proportion of the heterogenous “Others” ethnic group in those with cardiotoxicity compared to those without (28.1 vs. 6.8%; *p* < 0.001). The age of diagnosis and the cumulative anthracycline dose did not differ between the cardiotoxic and non-cardiotoxic groups; although, there was a trend toward more patients who were exposed to cumulative dose ≥250 mg/m^2^ (high dose) in the cardiotoxic group (46.9 vs. 31.3%; *p* = 0.07).

Twelve of these cardiotoxic patients (12/32, 37.5%) had symptoms of heart failure, whilst the rest were asymptomatic. Of these 12 patients with clinical heart failure (12/458, 2.6%), one had acute cardiotoxicity, five early cardiotoxicity, and six late cardiotoxicity; median LV FS was 20.7% (IQR 12.2–26.0%).

Among the 20 patients who were asymptomatic, i.e., subclinical cardiotoxicity (4.4%), 2 had acute cardiotoxicity, 7 early cardiotoxicity, and 11 late cardiotoxicity; median LV FS was 22.9% (IQR 20.3 – 26.3%). The cumulative anthracycline dose did not differ between clinical heart failure patients and those who were asymptomatic, 263 mg/m^2^ (IQR 150–300 mg/m^2^) vs. 187 mg/m^2^ (IQR 105–300 mg/m^2^); *p* = 0.31.

Table 3 shows the univariate regression analysis for risk factors of cardiotoxicity in the cohort. The only factor significantly associated with cardiotoxicity was the “Others” ethnicity with odds ratio 5.35 (95% CI 2.27–12.63), *p* < 0.001. Gender, age at diagnosis, and cumulative anthracycline dose were not significant risk factors. Table 4 describes the comparison of characteristics of participants with cardiotoxicity, between those of the “Others” and the main ethnic subsets. There were no significant differences between the two groups in terms of primary disease and traditional predictors of cardiotoxicity that could have accounted for the “Others” ethnic group being associated with cardiotoxicity.

**Table 3:** Univariate analysis for predictors of cardiotoxicity.

	**Odds Ratio**	**95% CI**	***p*-value**
Female Sex	0.71	0.33–1.54	0.39
**Race or Ethnicity**			
Chinese	0.50	0.24–1.04	0.06
Malay	1.19	0.47–3.01	0.71
Indian	0.27	0.03–2.05	0.21
Others	5.35	2.27–12.63	<0.001
Age at diagnosis, years	1.02	0.95–1.08	0.56
Follow-up duration	1.01	0.95–1.08	0.62
Cumulative doxorubicin equivalent dose	1.00	0.99–1.00	0.23
≥250 mg/m^2^	1.61	0.77–3.36	0.20
Radiation involving the heart	1.37	0.39–4.74	0.62

**Table 4:** Comparison of characteristics of participants with cardiotoxicity, between “others” and the main ethnic groups (Chinese, Malay, and Indian).

**Characteristics**	**Others (*n* = 9)**	**Main ethic groups (*n* = 23)**	***p*-value**
**Demographic**			
Sex			1.00
Female	3 (33.3)	7 (30.4)	
Male	6 (66.7)	16 (69.6)	
**Clinical**			
Diagnosis			
Acute Lymphoblastic Leukemia (ALL)	3	8	
Acute Myeloid Leukemia (AML)	3	5	
Neuroblastoma	0	5	
Osteosarcoma	1	3	
Lymphoma	1	1	
Ewing Sarcoma	1	0	
Hepatoblastoma	0	1	
Age at diagnosis, years	10.4 (3.9–12.5)	6.9 (2.9–13.1)	0.71
Cumulative doxorubicin equivalent dose, mg/m^2^	300 (120–300)	200 (150–360)	0.86
<250 mg/m^2^	4 (44.4)	13 (56.5)	
≥250 mg/m^2^	5 (55.6)	10 (43.5)	0.69
Radiation involving the heart	2 (22.2)	1 (4.3)	0.18

### Outcome of Patients With Cardiotoxicity

Half of the patients (6/12) with clinical heart failure had recovery of LV systolic function at 36.4 months (IQR 0.9–72.4 months) from the onset of cardiotoxicity. Of the remaining six, one had persistently abnormal LV systolic function, and five died (three from end stage heart failure and two from the primary disease). Sixty percent (12/20, 60%) of patient with subclinical cardiotoxicity had recovery of LV systolic function at 6.2 months (IQR 4.6–11.7 months) from the onset of cardiotoxicity. Of the remaining eight, one had persistently abnormal LV systolic function, and seven died (three from the primary disease, three from sepsis, and one from second malignant neoplasm).

**Figure 1:**
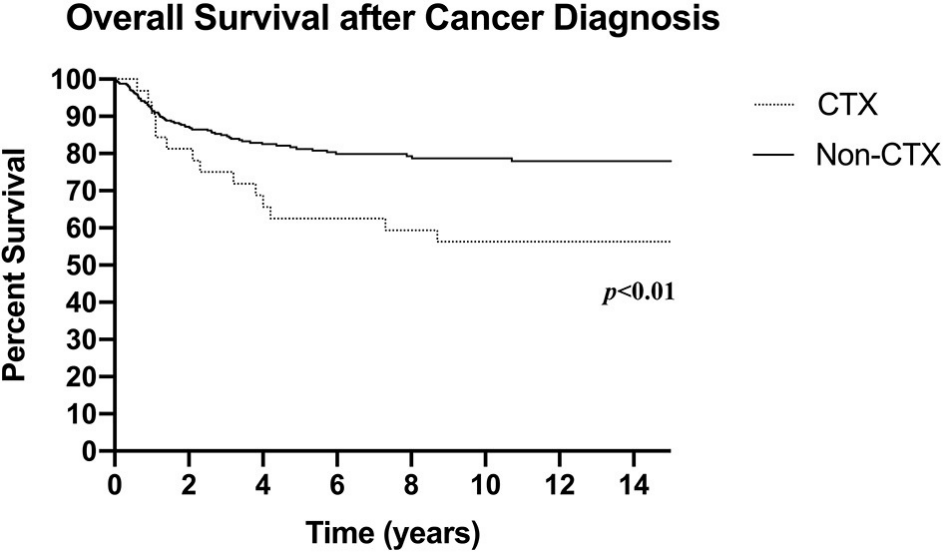
Overall survival after cancer diagnosis (CTX: *N* = 32, death *n* = 15; Non-CTX: *N* = 426, death *n* = 82; censored at last follow-up). CTX, Cardiotoxic Group; Non-CTX, Non-cardiotoxic Group.

Median follow-up was 3.9 years (IQR 2.1–9.5 years) for the entire cohort. During this time, 97 deaths occurred (21.2%) at median age 9.9 years (4.7–14.9 years). Total all-cause mortality (including death secondary to cardiotoxicity) in the cardiotoxic group was significantly higher compared to the non-cardiotoxic group (46.9 vs. 19.2%; *p* < 0.001; Figure 1).

Of the 15 (15/32, 46.9%) patients who died in the cardiotoxic group, 3/32 had died from end-stage heart failure, resulting in an anthracycline-induced cardiotoxicity mortality rate of 9.4%; 6 (18.8%) patients succumbed to their cancer or second malignancy, 5 (15.6%) deaths were due to septic complications, and the remaining 1 (3.1%) death was due to pulmonary edema secondary to renal failure from thrombotic microangiopathy. The median age of death within the cardiotoxic group was 11.8 years (IQR 7.2–18.0 years), with a median interval of 28 months (IQR 15.1–50.0 months) from cancer diagnosis.

## Discussion

Even though the cardiotoxic effects of anthracyclines are well-documented and widely reported, there is a paucity of data on its burden in the Asian populations. In the current study, we found that 7% of children treated with anthracyclines developed cardiotoxicity; the incidence of subclinical cardiotoxicity and anthracycline-related clinical heart failure were 4.4 and 2.6%, respectively. More than half of these (53%) presented with late cardiotoxicity. Although there was no one statistically significant ethnic predisposition to developing cardiotoxicity, we noticed that there was a higher proportion of non-Chinese/Malay/Indian (“Others”) in those with cardiotoxicity compared to those without (28.1 vs. 6.8%; *p* < 0.001). Patients with cardiotoxicity had a higher total all-cause mortality rate (46.9%) compared to those without cardiotoxicity (19.2%).

Although observed frequencies vary among studies, up to 57% of patients treated with anthracycline may develop echocardiographic abnormalities (5). This is widely attributable to differences in the definitions of echocardiographic abnormalities and study designs such as selection of non-random or non-representative subgroup of patients without the inclusion of the original patient cohort. Over the study period, we included consecutive children who were diagnosed with cancer and required anthracycline treatment as part of their chemotherapy protocol. In this original patient cohort, we have chosen to censor them at the date of the last echocardiographic study such that the echocardiographic outcome was measured at a time point which reflected the end of the study. At a short follow-up duration of 3.9 years, 7% of our cohort were found to have reduced LV systolic function (LV FS <28%), of whom 60% were asymptomatic (i.e., subclinical), and more than half of these (53%) occurred 1 or more years after completion of therapy. This is consistent with previous studies which showed that cardiac dysfunction from anthracycline chemotherapy frequently presents as late-onset cardiotoxicity which can manifest as an asymptomatic abnormality on cardiac imaging (Stage B heart failure), or as clinical heart failure (Stage C or D heart failure) (21–23). Notably, the relationship between subclinical cardiotoxicity and subsequent clinical heart failure is as yet unclear.

### Comparison With Existing Literature

There is a paucity of data from Asian populations on this subject matter in the literature; similar epidemiological studies in Asian populations are few and have smaller sample sizes compared to their Western counterparts. In Beijing, Hu et al. (16) described a 1.5% frequency of anthracycline-induced heart failure, which is lower than 2.6% in our cohort. However, this could owe itself to their smaller cohort size of 131 patients, with just 2 patients developing heart failure, over a shorter study period of 5 years. In South Korea, Kim et al. (24) described 5.2% (75/1453) of patients developing cardiotoxicity, which was defined as any cardiac abnormality including sudden cardiac death and early cardiotoxicity. However, the definition of cardiotoxicity was not clearly established, which made direct comparison with our current data challenging. Having said that, this South Korean study reported 2.1% incidence of symptomatic heart failure, which is similar to our current data of 2.6%. Kang et al. (24) in a separate South Korean study, described 7.3% incidence of dose-limiting cardiotoxicity in patients who were not treated with the cardioprotective drug, dexrazoxane. Incidentally, 2.7% (7/258) of their total cohort developed clinical congestive heart failure (25), which is similar to our data. Overall, there appears to be congruency of the incidence of heart failure in Asian populations.

On comparison with western populations, our heart failure incidence is similar to that of France and the UK at 2.8% (26) and Switzerland at 2.4% (27). On the other hand, two separate series from the USA reported divergent incidences of heart failure; Lipshultz et al. (6) described 10% developing acute heart failure whilst the Childhood Cancer Survivor Study (CCSS) reported 1.7% (21). Beyond symptomatic heart failure, the abovementioned Swiss study described the incidence of any cardiovascular disease at 14.3% while Getz et al. (28) in the USA described 12% cardiotoxicity amongst AML patients within a 5-year follow-up period (28), both of which were higher than our 7%. However, the Swiss study utilized self-reported data which could lead to inaccuracy of definition by study participants, hence a direct comparison may not be fully justified. Again, this highlights the differing definitions of cardiotoxicity used in different studies. Be that as it may, there was equipoise with regards to the incidence of symptomatic heart failure with our current data. The widely discrepant reported frequency of cardiotoxicity within the western literature suggested that the data may not translate to a similar response to anthracyclines in an Asian context.

### Assessment of Ventricular Systolic Function

Various studies have demonstrated the utility of early markers of ventricular remodeling or dysfunction using echocardiography, such as end-systolic wall stress, stress-velocity index (6), and thickness-to-dimension ratio (12). Although cardiac dysfunction may be defined using different assessment methods of systolic function and afterload, EF and FS are the two most commonly used parameters for assessment of ventricular function after anthracycline exposure. More advanced echocardiographic modalities (e.g., tissue Doppler imaging, speckle-tracking echocardiography with strain and strain rate) have since been applied. However, these modalities may be technically challenging for operators and may not be widely available in all institutions that follow CCS, especially in emerging regions in Asia. Institutionally, we have opted to use FS for monitoring of ventricular function because it is easily reproducible, can be monitored over time, and is a good surrogate measure for the impact of cancer treatment on myocardial systolic performance. Despite its limitations, FS is widely used in various chemotherapeutic protocols for the monitoring of heart function. In addition, it represents an “actionable” parameter for decision on pharmacologic interventions (e.g., angiotensin-converting enzyme (ACE) inhibitors, beta blocker), especially in the setting that the pre-emptive use of such medications in patients with evidence of ventricular remodeling but preserved ejection fraction being controversial (29, 30).

### Risk Factors for Cardiotoxicity

Interestingly, in our study, the only patient factor associated with higher risk of cardiotoxicity was the “Others” ethnicity (i.e., non-Chinese, non-Malay, and non-Indian). In multiracial Singapore, “Others” is a heterogeneous group of ethnicities outside the majority Chinese, Malay, and Indian ethnicities, predominantly comprising those of Caucasian descent, and in our study included foreign nationals (e.g., Filipino, Vietnamese, etc.). The racial breakdown of the cohort is similar to Singapore's population, with a Chinese majority with minority groups comprising of Malays, Indians, and Others (20), making it representative of the local population. While the elevated risk of this “Others” racial group may not be immediately intuitive owing to its heterogeneity, what is apparent in our study is that children from the main ethnic groups (Chinese, Malay, and Indian) showed no racial predilection to cardiotoxicity. There were also no significant differences between gender, age at diagnosis, cumulative anthracycline dose and irradiation between the patients of the main ethnic groups and “Others.”

Intriguingly, we could not confirm traditional predictors of cardiotoxicity to be significant risk factors of developing cardiotoxicity, of which previous studies have identified female sex, younger age at treatment, cumulative anthracycline dose, and radiation to a field that involved the heart as risk factors for anthracycline cardiotoxicity (5–7, 12). Notably, most of these studies were performed in the Western populations and are associated with late-onset LV dysfunction, which constitutes only 53.1% of our cohort. Conservatively put, evidence from studies conducted in Western populations may not be extrapolated to Asians because of genetic differences in drug responses and susceptibilities to the development of adverse, treatment-associated toxicities. Having said this, it may also be possible that the limited number of observed events in our study could have curtailed the statistical power to demonstrate the risk factors. Specifically, for cumulative anthracycline dose, despite the lack of statistical significance between symptomatic and asymptomatic cardiotoxicity (*p* = 0.31), the median cumulative anthracycline dose for symptomatic events was 40.6% higher than that of asymptomatic events.

### Pharmacoethnicity

The concept of pharmacoethnicity, which is broadly defined as both genetic and non-genetic ethnic diversity in drug responses and toxicities, is an emerging hypothesis proposed to explain inter-individual and inter-ethnic variations in drug responsiveness (31, 32). Different rates of chemotherapy-induced toxicities among Caucasian, African American, Asian, and Hispanic cancer populations have been attributed to variations in pharmacogenomics (28, 32–34). These variations include inherited genetic polymorphisms that render subgroups of cancer patients more susceptible to certain late effects. In this regard, genetic variations in anthracycline-induced chronic cardiotoxicity have been identified (17). Of these genetic variants, those responsible for doxorubicin transport and free radical metabolism may modulate the individual risk of cardiotoxicity development (35). Significantly, the distribution of different genotypes (CT/TT and CC) at the CYBA gene, which is involved in physiological free radical metabolism, differed in the sample of Chinese controls (proportion with variant allele = 21.1%), compared to Caucasians (54.1%) (35, 36). This genotypic difference was associated with significantly higher plasma high-sensitivity cardiac troponin T levels in those with CT/TT at CYBA gene than those with CC genotype, which suggests greater degree of injury to cardiac tissue in subjects with the variant allele (36).

Evolving data and our findings hence suggest that data from western literature may not accurately predict the incidence of cardiotoxicity in Asian patients. In fact, Asian patients could possibly either be at lower risk of developing anthracycline cytotoxicity or could develop less severe disease as our main ethnic groups (Chinese, Malay, and Indian) are Asian, contrasted to the Caucasian-majority ethnic group of “Others,” which was observed to be associated with higher risk of developing cardiotoxicity in our cohort. Therefore, the systematic characterization of treatment-related complications among Asian populations is expected to have important clinical implications in this new era of personalized medicine. Clinically, it is imperative for clinicians to understand these pharmacoethnic differences when counseling Asian patients and hence their subsequent cardiac surveillance and management.

### Prognosis and Mortality

While most studies have focused on understanding the risk of late-onset cardiotoxicity in patients who survived at least 5 years cancer-free following diagnosis (10, 12, 21, 37), our study sought to understand cardiotoxicity during and after initial chemotherapy for childhood cancers. We observed that most subclinical cardiotoxicity occurring during the initial treatment phase (acute or early cardiotoxicity) had subsequent recovery of heart function. This finding may suggest a favorable prognosis for patients with acute or early cardiotoxicity. Nevertheless, our patients who had developed cardiotoxicity had notably higher mortality compared to those with normal heart function, though majority (12/15, 80%) of the deaths in the cardiotoxic cohort were not due to heart failure itself. This correlation could suggest that the development of cardiotoxicity may predispose to a higher risk of developing fatal complications or through the reduction of functional reserve, regardless of development of overt clinical heart failure, both of which could exacerbate the severity of the primary disease. While most studies have confirmed the increased risk of cardiac death from anthracyclines (13–15), more research is required to ascertain the cause of increased all-cause mortality amongst cardiotoxic patients.

## Strengths and Limitations

The strength of this study was that it included the entire original cohort of children diagnosed and treated for their cancers in the recent era, during and after the initial chemotherapy; all of this done in a multiethnic Asian population. However, our study has important limitations which should be considered when interpreting the findings of our study. Firstly, this study has limitations inherent in a single-center retrospective database analysis with potential selection bias that could skew findings if a non-random population of patients were selected for analysis. Second, although we evaluated LVEDD in addition to LV FS, we did not have their values converted to z-scores based on body surface area. Indeed, chamber dilatation with preserved FS is a common observation in the pre-clinical presentation of cardiomyopathy. Finally, the frequency of surveillance after the conclusion of chemotherapy was at the discretion of each patient's treating oncologist, in the absence of an institutional protocol during the study period. Therefore, additional subclinical cardiotoxic events could have been missed, resulting in a falsely lowered reported incidence of anthracycline-induced cardiotoxicity. This could have thus affected our evaluation of cardiotoxicity risk factors due to the potential loss of number of events.

## Conclusion

This study provided a glimpse of the epidemiology of cardiotoxicity after anthracycline chemotherapy for childhood cancer in a multiethnic Southeast Asian population. Our study reaffirmed that freedom from symptoms does not ensure normal heart function and suggested that children with abnormal ventricular systolic function have higher risk of mortality compared to those with normal systolic function. The findings contribute to improved understanding of the local burden of disease in Singapore and in other Asian-majority populations, which may be pertinent to aid development of measures to prevent or reduce the risk of cardiac disease. Our findings also add to the pool of evolving data suggesting the importance of pharmacoethnicity in the development of cardiotoxicity. Clinically, it suggests the importance of having a systematic, periodic echocardiographic surveillance in these patients and better understanding of the Asian reaction to anthracyclines in order to achieve improved long-term patient outcomes especially in the impending era of personalized medicine. Further studies are needed to determine the optimal interval of such surveillance and clarify the most sensitive echocardiographic index for prediction of long-term cardiac outcome.

## Data Availability Statement

The raw data supporting the conclusions of this article will be made available by the authors, without undue reservation.

## Ethics Statement

The studies involving human participants were reviewed and approved by Singhealth Centralized Institutional Review Board (CIRB). Written informed consent from the participants' legal guardian/next of kin was not required to participate in this study in accordance with the national legislation and the institutional requirements.

## Author Contributions

VT participated in data analysis, statistical analysis, and writing of the manuscript. NC participated in data collection, data analysis, and writing of the manuscript. WA participated in data collection and approved the final manuscript. SM participated in performance of the research and approved the final manuscript. MC participated in performance of the research, reviewed the data, and approved the final manuscript. CC acted as the senior author, conceptualized and designed the study, reviewed the analyzed data, reviewed and revised the manuscript, and approved the final manuscript. All authors contributed to the article and approved the submitted version.

## Conflict of Interest

The authors declare that the research was conducted in the absence of any commercial or financial relationships that could be construed as a potential conflict of interest.
